# The Impact of Hearing Impairment on the Life Trajectories of Aboriginal Children in Remote Australia: Protocol for the Hearing Loss in Kids Project

**DOI:** 10.2196/15464

**Published:** 2020-01-15

**Authors:** Jiunn-Yih Su, Vincent Yaofeng He, Steven Guthridge, Sven Silburn

**Affiliations:** 1 Centre for Child Development and Education Menzies School of Health Research Charles Darwin University Casuarina Australia

**Keywords:** data linkage, hearing impairment, indigenous population, child development, primary schools, academic achievement, child maltreatment, juvenile delinquency

## Abstract

**Background:**

Previous studies have reported a high prevalence of chronic otitis media (OM) and hearing impairment (HI) in Aboriginal children in the Northern Territory (NT) of Australia. Children affected by these disorders are believed to be at increased risk for adverse outcomes in early childhood development, school attendance, academic performance, and child maltreatment and youth offending. However, to date, there have been no studies quantifying the association between HI and these outcomes in this population.

**Objective:**

This study will investigate the association between HI and the 5 outcomes in Aboriginal children living in remote NT communities.

**Methods:**

Individual-level information linked across multiple administrative datasets will be used to conduct a series of retrospective observational studies on selected developmental and school outcomes. The predictor variables for all studies are the results from audiometric hearing assessments. The outcome measures are as follows: Australian Early Development Census results, representing developmental readiness for school, assessed around 5 years of age; Year 1 school attendance rates; Year 3 school-based academic performance, assessed in the National Assessment Program—Literacy and Numeracy; incidence of child maltreatment events (including both notifications and substantiated cases); and incidence of a first guilty verdict for youth offenders. Confounding and moderating factors available for the analysis include both community-level factors (including school fixed effects, socioeconomic status, level of remoteness, and housing crowdedness) and individual-level factors (including maternal and perinatal health and hospital admissions in early childhood).

**Results:**

The study commenced in 2018, with ethics and data custodian approvals for data access and linkage. This has enabled the completion of data linkage and the commencement of data analysis for individual component studies, with findings expected to be published in 2019 and 2020.

**Conclusions:**

This study will provide first evidence of the impact of OM-related HI on the developmental, educational, and social outcomes of Australian Aboriginal children. The findings are expected to have significant implications for policy development, service design, and resource allocation.

**International Registered Report Identifier (IRRID):**

RR1-10.2196/15464

## Introduction

### Background

Children’s experiences during infancy and early childhood are critical to their health and educational and socioemotional well-being throughout their life course [1-5]. Through this period of rapid development, children are particularly sensitive to influences from their living environment [1,6]. At this age, the ability to hear is crucial to children’s normal development, as children learn to speak and communicate by imitating the sounds they hear, and hearing impairment (HI) is likely to reduce children’s exposure to sounds and voices and hamper their language development [7]. Consequently, delayed or impaired language learning can adversely affect children’s physical health, as well as social and emotional development [7-9].

Otitis media (OM) and the associated HI among Australian Aboriginal children has been extensively researched [10-19]. Community surveys conducted in Aboriginal children living in remote communities have consistently reported extremely high prevalence of OM, as high as 90% in some reports, which has persisted through decades of clinical and public health interventions [14,18-20]. Studies have also found that OM affects Aboriginal children early in life, is more severe, and persists for longer [7,10,21], with prevalence peaking at the young age of 5 to 9 months [15]. Among the affected children, if OM is left untreated or not treated adequately, it often progresses to chronic suppurative OM (CSOM) and tympanic membrane perforation, with mild to moderate conductive hearing loss [22-24]. The impact of HI on language development may be compounded in Aboriginal children, when English is a second or third language, but school-based teaching relies on English [22,25-28]. In this setting, it may be reasonably expected that OM-related HI may directly or indirectly impact children’s readiness for school and subsequent participation and performance at school, as well as their ongoing social and behavioral development through childhood and adolescence.

Although there is an abundance of the literature showing the correlations among child maltreatment, youth offending, early childhood development, school performance, and school attendance [29-33], to date, there has been no population-level investigation into the impact of HI on the long-term developmental, educational, and social outcomes of Aboriginal children. Furthermore, studies investigating the impact of OM (or its associated HI) on various aspects of early childhood development, including cognitive development, speech and language development, and educational outcomes, have produced equivocal results [7,34-36]. Several reasons have been proposed for such an inconclusiveness [22,34-37]. First, there are limitations in study design. Many studies have relied on surveys and have therefore been restricted by the inherent limitations of this design, as well as limited statistical power. On the other hand, the negative associations of OM with effusion (OME) and children’s later language development found in some prospective and randomized clinical trials still need to be verified with large-scale studies that can provide more precise population-level estimates. Second, the majority of these studies used clinical diagnoses of OM, such as OME [38] or chronic OM [39,40] as the independent variable, rather than a direct assessment of hearing. The uncertainty surrounding the presence and degree of HI when OM is used as the predictor variable compromises the validity of the findings. Third, many studies have not controlled for known confounding or moderating variables, such as maternal education and socioeconomic status [41-44]. Finally, there has been a lack of population-level audiometric assessment data to report population-level outcomes.

### Objectives

**Figure 1:**
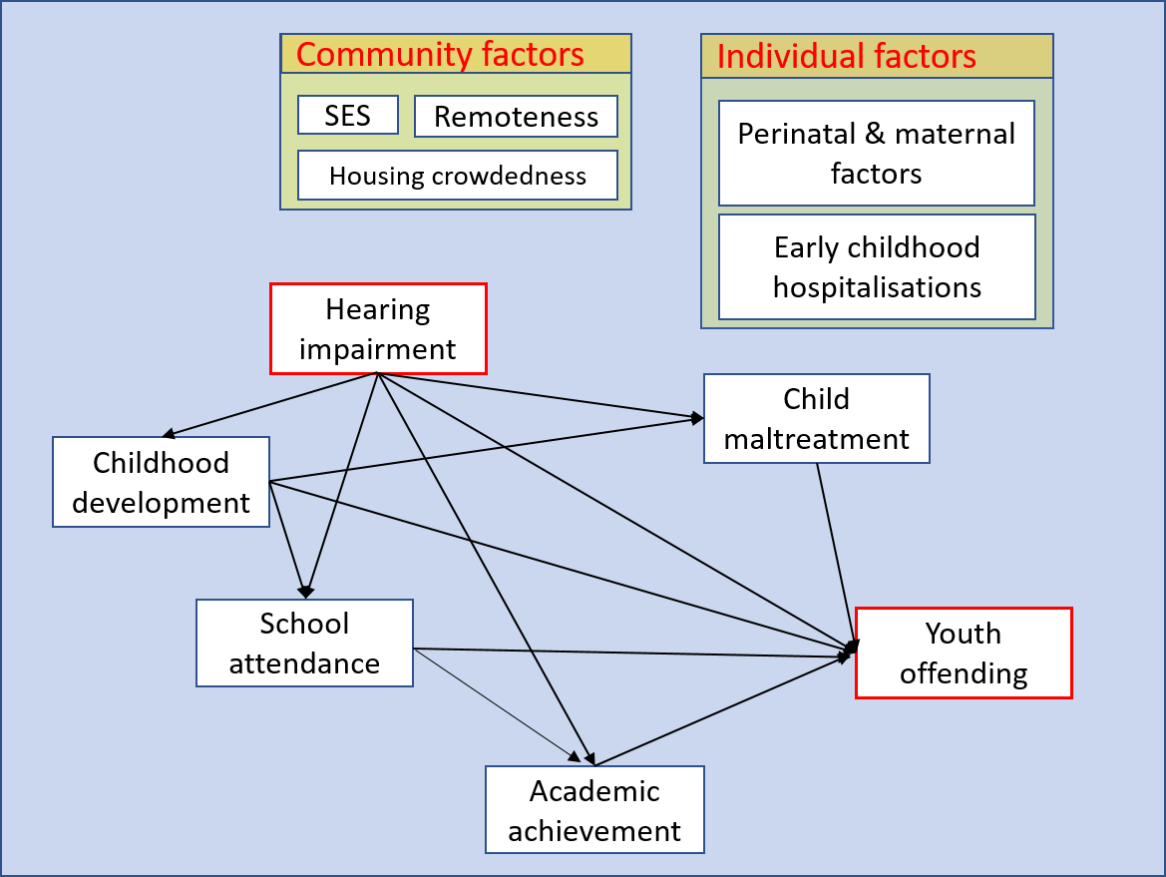
Conceptual diagram indicating the pathways of influence of hearing impairment on life trajectories and the interplay between them (SES: socioeconomic status).

To address this research gap, the Hearing Loss in Kids (HeLoKids) Project aims to apply a life-course epidemiological approach [45] to investigate the association between HI and developmental, educational, and social outcomes in Aboriginal children living in remote Northern Territory (NT) communities. By using linked administrative datasets containing longitudinal unit-record data for health, education, child protection, and youth justice systems, as well as hearing assessment results, this study is able to examine the selected outcomes while controlling for a wide range of potential confounders and moderators, as illustrated in the conceptual model in Figure 1.

Specifically, the study will undertake a series of studies that test the following hypotheses:

1. HI is independently associated with poorer developmental outcomes at the time of entry into full-time school (aged about 5 years).
2. HI is independently associated with lower school attendance rates in Year 1 (aged about 6-7 years).
3. HI is independently associated with poorer academic achievements in Year 3 (aged about 8 years).
4. HI is independently associated with higher risk of child maltreatment.
5. HI is independently associated with higher risk of youth offending.

## Methods

### Setting

The NT is located in the north and central part of Australia, with an area of 1,352,176 square kilometers. It has the smallest total population among Australian states and territories, with an estimated resident population of 247,300 in 2018 [46], and the highest proportion of Aboriginal people in the total population (about 30%, compared with 3% for Australia) [47]. The NT also has the highest proportion of the total population living in remote and very remote areas (40.4% in 2017), as measured using the Accessibility and Remoteness Index of Australia (ARIA+) [48,49]. NT Aboriginal people have also been reported to be socioeconomically disadvantaged, as measured with the Socio-Economic Indexes for Areas, based on aggregated Australian population census data on social and economic status [50]. In addition to the high prevalence of OM, NT Aboriginal children have lower school attendance and retention, lower academic achievement, higher rates of reported child maltreatment, and higher rates of youth offending than their non-Aboriginal counterparts [51-55].

### Design and Linkage Process

The HeLoKids Project will be conducted as a series of retrospective observational cohort studies to examine the association between HI and a range of developmental, educational, and social outcomes. These studies will utilize administrative datasets held within an extensive repository of deidentified unit-level information for NT children, which has been incrementally developed since 2009 [56]. SA NT DataLink (a South Australia [SA] and NT partnership) [57] completed the linkage of datasets collated in the repository. The data linkage process involves individual data custodians preparing a dataset containing identifying information only and then forwarding the dataset to SA NT DataLink. SA NT DataLink then combines the datasets from multiple custodians and links records for the same individual, which are present in the various datasets, and creates an anonymized project-specific linkage key for each individual. SA NT DataLink then adds the linkage key to the original datasets and returns each dataset to the respective data custodian. The data custodian creates a research dataset by combining the linkage keys with approved research variables and removing identifying information. The research dataset, created by each custodian, is then forwarded to the research team. The research team is able to use the linkage keys to merge information on individuals from multiple datasets to create the datasets necessary for analysis.

### Remote Hearing Assessment Dataset

The Remote Hearing Assessment (RHA) dataset contains middle ear examination and audiometric assessment information collected by the NT Outreach Hearing Health Program [58]. This program is an Australian Government funded specialist ear and hearing health outreach service, available to all NT Aboriginal children and young people, aged under 21 years, most of whom live in remote communities.

The NT Outreach Hearing Health Program uses pure tone audiometry to assess children’s hearing ability and determine the type (sensorineural, conductive, mixed, or indeterminate) and level of hearing loss. The level of hearing loss of an ear is calculated by taking the average of the thresholds of hearing (as deviation from normal threshold, in decibels Hearing Level—dB HL) for 3 frequencies: 500 Hz, 1000 Hz, and 2000 Hz. The results for each ear are classified as either normal or 1 of 4 ordinal categories of hearing loss, namely mild (16-30 dB HL), moderate (31-60 dB HL), severe (61-90 dB HL), and profound (91 dB HL or greater). This relatively conservative classification of hearing loss was chosen over the more widely used World Health Organization classification, as it is deemed more suitable for children aged under 15 years [58,59]. Further details on this classification of hearing loss are available elsewhere [58]. The clinical assessment for each ear is also recorded and classified under the following categories: acute OM (AOM), AOM with perforation, CSOM, dry perforation, Eustachian tube dysfunction, increase in middle ear pressure, OME, normal, and others.

The dataset provided to this study includes information collected from 2007 to 2015, for over 8000 children and young people aged between 0 and 21 years, which is about 22% of the NT Aboriginal population in this age group. Children and young people access these services through referral by primary care services. From January 2013, access to these services was prioritized according to clients’ need for service. This means that the children and young people whose records are captured in the RHA dataset are not a random sample of the general population, and the information is likely to be biased toward those with abnormal middle ear examination and hearing difficulties.

### The Study Cohort

The study cohort comprises NT-born Aboriginal children, with hearing assessment results in the RHA dataset and with a residential location classified as *remote* or *very remote* on the basis of ARIA+ [48]. Further inclusion and exclusion criteria will vary among the component studies of this study, depending on the respective requirements. In principle, as the data analysis will be based on a complete-case analysis, only children with data in the selected dependent, outcome, and control variables will be included. Children who have had surgical treatment for OM before the age of 4 years will be excluded, as the surgery could alter the impact of HI during the early childhood period. This will be done by searching in another linked administrative dataset, the NT Hospital Separations Dataset, and excluding children admitted before the age of 4 years, with any diagnosis code plus any procedure code related to OM and its treatment (Table 1).

**Table 1:** Otitis media—related hospital admission diagnoses and surgical procedures used to exclude children who had received surgical treatment, with International Classification, 10th Revision-Australian Modification codes.

ICD-10AM^a^ Code		Diagnosis/procedure
**Diagnosis**		
	H65	Nonsuppurative otitis media
	H66	Suppurative and unspecified otitis media
	H72	Perforation of tympanic membrane
**Procedure**		
	41527-00	Myringoplasty, transcanal approach
	41530-00	Myringoplasty postaural or endaural approach
	41533-01	Myringoplasty with atticotomy
	41542-00	Myringoplasty with ossicular chain reconstruction
	41551-00	Mastoidectomy by intact canal wall technique with myringoplasty
	41554-00	Mastoidectomy by intact canal wall technique with myringoplasty and ossicular chain reconstruction
	41560-00	Modified radical mastoidectomy with myringoplasty
	41560-01	Radical mastoidectomy with myringoplasty
	41563-00	Modified radical mastoidectomy with myringoplasty and ossicular chain reconstruction
	41563-01	Radical mastoidectomy with myringoplasty and ossicular chain reconstruction
	41626-00	Myringotomy, unilateral
	41626-01	Myringotomy, bilateral
	41632-00	Myringotomy with insertion of tube, unilateral
	41632-01	Myringotomy with insertion of tube, bilateral
	41635-01	Excision of lesion of middle ear with myringoplasty
	41638-01	Excision of lesion of middle ear with myringoplasty and ossicular chain reconstruction
	41789-00	Tonsillectomy without adenoidectomy
	41789-01	Tonsillectomy with adenoidectomy
	41801-00	Adenoidectomy without tonsillectomy
	90114-00	Other procedures on eardrum or middle ear

^a^International Classification, 10th Revision-Australian Modification.

### Dependent Variables

For this study, the term “hearing loss” (HL) is used for a single ear to refer to a certain degree of loss of hearing ability, whereas “hearing impairment” (HI) is used for an individual’s state of hearing loss in both ears. The dependent variables used across all component studies will be HI, and its values will be derived from the hearing assessment data contained in the RHA dataset. It will be an ordinal variable comprising the following categories:

- Normal hearing: normal audiometry results in both ears.
- Unilateral hearing loss: normal in one ear and any degree of hearing loss in the other.
- Mild HI: mild hearing loss in the better hearing ear.
- Moderate-or-worse HI: moderate-or-worse hearing loss in the better hearing ear.

Although OM tends to develop early in life in NT Aboriginal children and be persistent and often asymptomatic [19,21], a child may not have a hearing assessment until the child is older, when both the diagnosis of OM and hearing assessment become easier. As a result, a proportion of children may not be tested until after the outcome event for the study. There are also a proportion of children with multiple records of hearing assessment in the RHA database. For the purpose of this study, we assume the results for the first hearing assessment of a child, at any age, are representative of the long-term hearing level for that child.

### Outcome Measures

This study aimed to investigate the impact of HI on the 5 aspects of life-course trajectory, listed in the following sections.

#### Early Childhood Development and School Readiness

The Australian Early Development Census (AEDC) is a cross-sectional national census of all children at the time of school entry, and it has been conducted triennially since 2009 [60]. The AEDC involves classroom teachers assessing children, aged about 5 years, across 5 domains of early childhood development, associated with readiness for school learning, namely physical health and well-being, social competence, emotional maturity, language and cognitive skills (school based), and communication skills and general knowledge. The score for an AEDC domain is marked on a scale of 0 to 10, with higher scores indicating higher levels of development. A score falling below the 10th percentile of the national AEDC population indicates a *developmentally vulnerable* result on that domain [61].

Data on the outcomes of early childhood development will be accessed from the AEDC dataset, one of the linked administrative datasets used in this study. The AEDC data available for this study contain data from the censuses held in 2009, 2012, and 2015. The first type of outcome variables is directly available in the AEDC dataset and are the dichotomous results of developmental vulnerability for individual domains and the summary measure of *being developmentally vulnerable on two or more domains* (DV2). Children assessed as vulnerable on 2 or more domains are considered to be at higher developmental risk, and they generally require additional support to progress through early schooling.

The second type of outcome variables is the domain scores, which are continuous variables. These outcome measures will be used because of the fact that a high proportion of children in our study cohort have been assessed as *vulnerable*, using the dichotomous outcome variables [61], and the use of continuous domain scores is anticipated to be more sensitive in detecting differences among groups. We will use the scores for individual domains in domain-specific analysis and the sum of the scores of the 5 domains as a general indicator of children’s overall readiness for school learning.

#### School Attendance in Year 1 of Primary School

The outcome measure for this component will be children’s annual school attendance rate for Year 1 (aged approximately 6-7 years). Year 1 is chosen, as it is the first year of compulsory school attendance in the NT and as, in the study population, school attendance rates decrease with increasing year level. The data source will be the School Attendance dataset, provided by the NT Department of Education, which contains records of enrollment and daily attendance status for students studying in NT Government schools for the period 2005 to 2016. The school attendance outcome will be expressed as the number of school days attended per year, with approximately 200 school days available per year.

#### Academic Performance in Year 3 of Primary School

The outcome measure for children’s academic performance in early years of primary school will be taken from the National Assessment Program–Literacy and Numeracy (NAPLAN) dataset. NAPLAN is an annual national assessment program, which commenced in 2008, and is undertaken by all students in Australia in years 3, 5, 7, and 9 (aged approximately 8, 10, 12, and 14 years, respectively) [62]. NAPLAN includes separate tests for numeracy, reading, writing, and language conventions (which include spelling, grammar, and punctuation). The test contents are developed to include specific considerations for Indigenous education, English as a second language, and special needs education [63]. The tests are designed to assess students’ understanding of the core elements of the national curriculum and to assist schools and teachers to identify students who may not have learned the skills required to progress academically. The raw score for each test is converted into a scaled score out of 1000. Data were available from 2008 to 2016. The outcome measures for this study will be the scale scores for the 5 NAPLAN domains for Year 3. The selection of Year 3 optimizes the study cohort by avoiding both the declining school attendance and declining participation in NAPLAN assessments in later years.

#### Child Maltreatment

The data source for this study is the NT Government Child Protection dataset, which is a statutory data collection of child maltreatment—related contacts with child protection services. The research dataset will include information on all notifications (reports), substantiated cases, and episodes of out-of-home care. The outcome measures for this study will be the cumulative incidence of first notification of child maltreatment and the cumulative incidence of first substantiated case of child maltreatment. The time to event will be calculated from date of birth to the earliest of the following: date of each event of interest, date of death, the last observed date in the linked datasets, or the designated end of follow-up date for the study. Data are available from 1999 to 2017.

#### Youth Offending

The Youth Justice dataset was provided by the NT Department of the Attorney-General and Justice, and this contains records of youth offending in the NT. The outcome measure for this study will be the cumulative incidence of first episode of a proven guilty offence within the youth justice system. The starting point for follow-up will be the 10th birthday, which is the minimum legal age of criminal responsibility, and the follow-up time will be calculated to the date of first offence or censored at the date of death, the last observed date in the linked datasets, or the set endpoint for follow-up of this study. Data are available for the period 1997 to 2017.

### Control Variables

The selection of control variables will vary between studies and the availability of the relevant variables in the linked datasets. We will draw reference from published literature in selecting control variables known to have confounding or moderating effects on the association being investigated. Individual-level control variables will be retrieved from the linked health, education, child protection, and justice datasets, including maternal and perinatal factors from the Perinatal Registry dataset (a statutory collection containing information for all births in the NT), as well as hospital admission diagnoses from the NT Hospital Separation Dataset. Community-level factors will include relative level of geographic remoteness (as measured with ARIA+), housing crowdedness (as measured with average household size and average persons per bedroom in the community), and socioeconomic disadvantage (as measured with the Index of Relative Socio-Economic Disadvantage) [48,50,64]. Aggregated data on these factors will be combined with the linked administrative datasets, using the relevant location variable for each study (either community of residence or school location).

### Statistical Analysis Plan

For each component study, statistical analyses will start with descriptive statistics to examine the distribution of key demographic, control, predictor, and outcome variables in the study cohort. The approach of complete-case analysis will be adopted in regression analysis. As this approach may lead to exclusion of participants with missing data, we will include comparisons of the study cohort and those excluded from analysis using suitable statistical methods (z-test, *t* test or chi-square analysis) to assess the differences between the 2 groups. When the groups are found to be statistically different on any number of included variables, we will include discussion of the implications of these differences on the validity of the study’s findings.

The dependent variable, HI, is structured as an ordinal variable, and we anticipate a *dose-response* relationship between the levels of HI and the outcome variables. In all regression analyses, the investigation of associations will use *normal hearing* as the reference category.

The study investigating the impact on AEDC outcomes will use logistic regression for dichotomous outcomes and linear regression on continuous ones. In the studies investigating the impact of HI on Year 1 attendance rates and NAPLAN results, multivariate linear regression models will be fitted to estimate the association between HI and the school-based outcome measures, and the models will include school-fixed effects to control for the average observed and unobserved differences in schools and students of the schools. In the studies examining the association between HI and child maltreatment and between HI and youth offending, multivariate survival analysis will be used by fitting Cox proportional hazard models.

In these component studies, a parsimonious model building strategy will be adopted in the regression modeling process. Univariate regression analysis will first be carried out to select variables with *P* value<.25. These and all possible 2-way interaction terms will be fitted in the multivariate regression model–building process. Variables deemed to be key confounders or moderators selected on the basis of past studies will be retained throughout the model-building process.

The final part of the study will investigate the interplay of key early childhood factors known to be associated with youth offending. It will examine how and to what extent the outcome of youth offending in NT Aboriginal children is shaped by the health, developmental, educational, and social factors investigated in this study, including HI, AEDC outcomes, Year 1 attendance rates, Year 3 NAPLAN results, and child maltreatment. The analysis will also examine the influence from key demographic factors relevant to children’s family and school learning circumstances. Structural equation modeling (SEM), using path analysis methods, will be utilized to quantify the relative impact of these factors on the outcome of youth offending. Distributional assumptions will be assessed to identify any outliners. The model will examine the direct pathway through which HI is associated with youth offending; it will also include the indirect pathways by which AEDC outcomes, Year 1 school attendance, and Year 3 NAPLAN results act as mediators in the direct pathway. In the SEM analysis, the *maximum likelihood with missing values* convergence method will be used to relax the requirement for each child to have complete data, as it is expected that the number of children will be reduced after linking multiple datasets. Relevant model fit indices will be reported for each pathway, including *P* value, model chi-square, goodness of fit index, p of close fit, comparative fit index, the root-mean-squared error of approximation, and the standardized root mean square residual. All statistical analyses will be performed using Stata for Windows, Version 15 (StataCorp).

## Results

The study commenced in 2018. Ethics clearance and data custodian approvals for data access and linkage have been obtained. This has enabled the completion of data linkage and the commencement of data analysis for individual component studies. Findings are expected to be submitted for publication in peer-reviewed journals in 2019 and 2020.

## Discussion

### Principal Findings

The availability of the RHA dataset for data linkage with a wide range of health, education, child protection, and youth justice datasets has provided an unprecedented opportunity for investigating the impact of HI on life-course trajectory of Aboriginal children and young people. The hearing assessment results obtained through pure tone audiometry for a large cohort of remote-dwelling Aboriginal children have enabled the direct use of HI as the predictor variable in this study, instead of using its precursor or causative condition, OM, which may introduce an unknown level of misclassification. It is expected that this study will provide the first comprehensive evidence for the impact of HI on these various measures of the life-course trajectory in the study population. Furthermore, the comparatively large sample and the use of raw test scores of AEDC and NAPLAN as continuous variables will provide a robust statistical power, which will increase the likelihood of detecting significant association with the selected outcomes. In each of the component studies, we will discuss sources of potential bias. One example is that children included in the RHA database are not a random sample of remote Aboriginal children in the NT, with a risk of selection bias. A second source of potential bias is the use of complete-case analysis. We will also discuss the likely direction of potential bias and the extent to which they may affect the validity of our results.

### Conclusions

The findings of this study will have significant implications for government departments and health and social service providers in policy development, service design, and resource allocation. The results can also be used as baseline measurements for monitoring interventions aimed at improving the ear and hearing health of Aboriginal children, as well as their developmental, educational, and social outcomes. The use of a linked administrative dataset in this study will further illustrate the utility of data linkage research methods for informing comprehensive service planning and evaluation.

## Abbreviations

AEDC: Australian Early Development Census
AOM: acute otitis media
ARIA+: Accessibility and Remoteness Index of Australia
CSOM: chronic suppurative otitis media
dB HL: decibels hearing level
HeLoKids: Hearing Loss in Kids
HI: hearing impairment
NAPLAN: National Assessment Program–Literacy and Numeracy
NT: Northern Territory
OM: otitis media
OME: otitis media with effusion
RHA: Remote Hearing Assessment
SA: South Australia
SEM: structural equation modeling
